# Oral health of children and adolescents with or without attention deficit hyperactivity disorder (ADHD) living in residential care in rural Rhineland-Palatinate, Germany

**DOI:** 10.1186/s12903-019-0948-5

**Published:** 2019-11-25

**Authors:** Vicky Ehlers, Angelika Callaway, Sophia Wantzen, Michael Patyna, James Deschner, Birgül Azrak

**Affiliations:** Department of Periodontology and Operative Dentistry, University Medical Centre of the Johannes Gutenberg University, Augustusplatz 2, 55131 Mainz, Germany

**Keywords:** ADHD, Oral health status, Residential care setting, Children and adolescents

## Abstract

**Background:**

Attention deficit hyperactivity disorder (ADHD) is defined as childhood neurobehavioural disorder. Due to short attention span, oral hygiene and dental treatment of such individuals can be challenging. Aim of this study was to evaluate the oral health of children and adolescents with and without ADHD living in residential care in rural Rhineland-Palatinate, Germany.

**Methods:**

Included in the study were 79 participants (male/female:58/21, age 9–15 years) living in residential care: 34 participants with ADHD and 45 participants without ADHD (control). Oral examination included the following parameters decayed, missing, filled teeth in the primary dentition (dmft), decayed, missing, filled surfaces/teeth in the secondary dentition (DMFS/DMFT), approximal plaque index (API), bruxism and orthodontic treatment. Additionally, oral hygiene, last dental visit and treatment performed, and dietary habits were assessed by questionnaire.

**Results:**

There were no significant differences in dmft, API, bruxism and oral hygiene habits between groups. However, participants with ADHD tended to have higher DMFS/DMFT values than the control group. Ongoing orthodontic treatment was found more often in the control group. The ADHD group tended to consume acidic/sugary beverages and sweet snacks more often than the controls. Different treatments (control visit/prophylaxis, dental therapy, orthodontic treatment) were performed at the last dental visit in the two groups.

**Conclusions:**

Within the limitations of this study**,** oral health was similar in children and adolescents with or without ADHD from the same residential care setting. Parents/guardians need instructions for better supervision of oral hygiene and dietary habits to improve the poor oral health of children with or without ADHD.

## Background

Attention deficit hyperactivity disorder (ADHD) is one of the most common neurobehavioural disorders of childhood, which can persist into adolescence and adulthood. The prevalence of ADHD ranges from 2 to 18% [1], with a prevalence below 5% in Europe [2]. ADHD occurs more often in boys than in girls [2]. The clinical presentation of ADHD varies and can be classified according to the International Statistical Classification of Diseases and Related Health Problems 10th Revision (ICD-10) [3] in disturbance of activity and attention, hyperkinetic conduct disorder and other hyperkinetic disorders. The characteristic features of ADHD include impulsivity, hyperactivity, and a short attention span. Also, difficulties with listening, compliance, and socialising are seen in children with ADHD. This can have consequences for oral health and dental treatment of these children, including therapy after traumatic dental injury [4].

It has been shown that children with ADHD have overall a poorer oral health status [5, 6] and oral hygiene attitudes [7] and higher plaque indices [8, 9] than those without ADHD. Higher caries prevalence in the ADHD group could be caused by their less effective tooth brushing due to their short attention span and difficulties to stay focused [5]. In addition, children with ADHD can have difficulties with performing various motor skills [10].

Xerostomia is mentioned as one of the adverse orofacial side effects of most drugs, which are commonly used for pharmacological treatment of ADHD [11]. Lower unstimulated salivary flow rates were found in participants with ADHD with [8, 12] or without [8] medication.

Concerning dietary habits, it was shown that there was a trend for more children with ADHD to eat sweet snacks [9, 13] or drink acidic/sugary beverages [13] between meals compared with the control group. Higher hyperactivity/inattention scores were positively correlated with poor oral health and consumption of cariogenic food [14].

Regarding dental caries, Kohlboeck et al. [15] reported a positive correlation of non-cavitated caries lesions with the presence of hyperactivity/ inattention in children. Children for whom their parents/guardians reported the presence of signs of inattention and hyperactivity had a higher risk for dental caries [16]. It was shown that children with ADHD had a significantly higher total enamel caries experience when compared to controls [17]. In some studies [5, 18], in children with ADHD significantly higher DS scores were found compared with the controls, whereas other studies [7, 13] did not find significant differences in DS/DMFS scores between children with or without ADHD.

Due to the characteristic features of ADHD, oral hygiene and dental treatment of children and adolescents with ADHD can be a challenge. The aim of the study was to assess oral health parameters of children and adolescents with or without ADHD living in residential care under supervision of specially trained caregivers. The null hypothesis was that oral health parameters of children and adolescents with or without ADHD, living in the same residential care setting under similar conditions, will not be different.

## Methods

### Participants

This study was conducted among children and adolescents, living in residential care in a rural area in Rhineland-Palatinate, Germany, due to familial problems, including loss of a parent or domestic violence. The children only spend a limited time in the residential care setting, where they live in small groups together with guardians. They ate two of their meals (breakfast and a cold meal in the evening) in this setting, while the main meal at lunch time was either consumed in the school cafeteria or in the residential care setting. Oral hygiene measures twice daily after breakfast and before bedtime were supervised by the guardians. The children went for dental consultation visits to various dentists, none of whom was specially trained for treating children with special needs. Some of the children and adolescents were permitted to spend the weekend one to three times per month in their family home. All participants were examined to assess oral health conditions as well as oral hygiene and dietary habits. This study was part of a group prophylaxis program for children and adolescents in Rhineland-Palatinate, Germany, where yearly dental examinations are conducted. Prior written consent was obtained from all parents or guardians of participating children and adolescents; participation in the study was voluntary. This study comprised two groups of children and adolescents: one group with children and adolescents with ADHD, and as control group children and adolescents without ADHD. The examiner was informed about which participants belonged to the ADHD group only after the study was completed. However, in most cases, due to the erratic behaviour of the ADHD participants, the diagnosis could be suspected.

### Inclusion and exclusion criteria

The age of the children and adolescents with ADHD living in this residential care setting ranged from 9 to 15 years, with one child aged 6 years. The age of the children and adolescents without ADHD ranged from 8 to 18 years. To match the two groups for age and sex, only participants aged 9–15 years and both sexes were included in this study. The ADHD group included children and adolescents with diagnosed ADHD by their paediatricians according to the classification ICD-10 [3] F90.0 (disturbance of activity and attention), F90.1 (hyperkinetic conduct disorder) and F90.8 (other hyperkinetic disorders). The type of pharmacological treatment for ADHD was documented. The control group included healthy children and adolescents living in the same residential care setting under the same conditions. The exclusion criteria comprised an intelligence quotient (IQ) *<* 50, eating disorders and missing written consent for participation.

### Clinical examination

All participants of the study were examined by the same investigator (SW), who was previously trained by a paediatric dentist (BA). Dental examinations included decayed, missing, filled surfaces/teeth in the secondary dentition (DMFS/ DMFT), decayed, missing, filled teeth in the primary dentition (dmft), and API (approximal plaque index %) according to Lange et al. [19]. Only cavitated lesions were included in the D component. The gingival tissues were visually assessed for absence (healthy conditions) or presence of supragingival plaque, reddening or ulceration (signs of gingivitis). All permanent teeth were visually evaluated for the presence of excessive tooth wear (bruxism). Ongoing or needed orthodontic treatment according to Genzel [20] was documented. Simplified examinations were conducted on site in rooms of the residential care setting, using a dental mirror and probe (EXS9, Hu-Friedy, Chicago, IL, USA) and a dental LED head-lamp with 40,000 lx. For assessment of API, plaque disclosing tablets were used (Mira-2-Ton®, Hager & Werken, Duisburg, Germany). The four oral hygiene scores (OH) were defined as follows: OH-1: API ≤ 25% (excellent); OH-2: 25% < API ≤ 50% (good); OH-3: 50% < API ≤ 75% (fair); OH-4: 75% < API ≤ 100% (poor) [21].

### Oral hygiene and dietary habits

A comprehensive questionnaire was developed and used in this study to assess oral hygiene and dietary habits (Additional file 1). All possibilities for oral hygiene were listed in the questionnaire. In addition, for each meal and for the time between meals, the possibilities were listed in an impartial manner to allow for honest answers. The children and adolescents themselves were asked to complete the questionnaires; in cases with signs of dyslexia their caregivers were available to assist them. Questions included tooth brushing habits, fluoridation, time passed since the last visit to the dentist and type of treatment performed during the visit (control visit/prophylaxis, dental therapy, orthodontic treatment). Information requested about dietary habits included type of beverage, frequency of intake of acidic/sugary beverage, type of sweets eaten between meals and frequency of consumption of sweet snacks.

### Statistical analysis

The data were analysed with the statistical software SPSS (version 23.0; SPSS Inc., Chicago, IL, USA). For categorical variables (gender, type of medication, ADHD classification, answers to topics from the questionnaire, presence or absence of bruxism, orthodontic treatment ongoing/needed/not needed, time passed since the last visit to the dentist and type of treatment performed during the visit) only absolute and relative frequencies are given as descriptive information. For numerical continuous and discrete variables (age, months in institution, API, DMFS/DMFT/dmft) mean, standard deviation (SD), median, minimum (min), maximum (max), and frequencies were calculated. Comparisons between ADHD group and control group for categorical variables were performed using the Chi-square test; for comparisons of numerical variables the non-parametric Mann-Whitney *U*-Test for two independent samples was used. *P* values of *<*0.05 were considered to be statistically significant.

## Results

### Study population characteristics

In this study, a total of 79 children and adolescents was examined, 34 of them with ADHD and 45 without ADHD, which served as control group. Table 1 shows the characteristics of the study population. In the ADHD group the majority (27 participants; 79.4%) was male and only seven participants (20.6%) were female. The control group consisted of 31 male (68.9%) participants, 14 were female (31.1%). The mean age in the ADHD group was 12.4 ± 1.9 years and in the control group the mean age was 12.8 ± 1.9 years. Methylphenidate (41.2%) was the most common medication for ADHD. The other types of medication included methylphenidate and risperidone (23.5%), amphetamine and risperidone (5.9%) as well as atomoxetine and risperidone (5.9%). However, almost a quarter (23.5%) of the participants with ADHD received no medication. The children and adolescents from the ADHD group lived in residential care with a mean of 21.3 ± 15.7 months, and those of the control group with a mean of 25.2 ± 30.3 months. Thirteen participants (38.2%) had the subtype F 90.0 (disturbance of activity and attention), the majority (20 participants; 58.8%) had the subtype F90.1 (hyperkinetic conduct disorder), and only one participant (2.9%) had the subtype F90.8 (other hyperkinetic disorders).

**Table 1:** Characteristics of the study population

	Children and adolescents with ADHD (study group) (*n* = 34)	Children and adolescents without ADHD (control group) (*n* = 45)
Gender:		
male	27 (79.4%)	31 (68.9%)
female	7 (20.6%)	14 (31.1%)
Age (years):		
mean ± SD	12.38 ± 1.89	12.76 ± 1.91
median/minimum/maximum	12/9/15	13/9/15
Medication for ADHD:		
• methylphenidate	14 (41.2%)	0
• methylphenidate and risperidone	8 (23.5%)	0
• amphetamine and risperidone	2 (5.9%)	0
• atomoxetine and risperidone	2 (5.9%)	0
• no medication	8 (23.5%)	45 (100%)
Months in residential care:		
mean ± SD	21.29 ± 15.74	25.24 ± 30.27
median/minimum/maximum	19/0.7/62	17/1/149
ADHD classification (ICD-10):		
F 90.0	13 (38.2%)	N/A
F 90.1	20 (58.8%)	N/A
F 90.8	1 (2.9%)	N/A

### Oral health

In the ADHD group 12 of 34 participants had primary teeth with a mean dmft of 0.33 ± 0.89 (median = 0, min = 0, max = 3) and in the control group 15 of 45 participants had primary teeth with a mean dmft of 0.47 ± 0.83 (median = 0, min = 0, max = 3). There was no statistically significant difference between the two groups (*p* = 0.39).

The permanent teeth were assessed for DMFS and DMFT (Table 2). The mean DMFS of the participants with ADHD was 3.91 ± 9.17 (median = 1, min = 0, max = 51) and the mean DMFS of the participants without ADHD had a value of 1.98 ± 2.71 (median = 1, min = 0, max = 13). In the ADHD group the mean DMFT was 1.91 ± 3.02 (median = 1, min = 0, max = 14) and the control group had a mean DMFT of 1.44 ± 1.79 (median = 1, min = 0, max = 7). There were no statistically significant differences between the two groups concerning both DMFS (*p* = 0.722) and DMFT (*p* = 0.983).

**Table 2:** Oral health parameters, orthodontic treatment, and oral hygiene habits in the study population

	Children and adolescents with ADHD (study group) (*n* = 34)	Children and adolescents without ADHD (control group) (*n* = 45)	*P*-value
DMFS	3.91 ± 9.17	1.98 ± 2.71	0.722
DMFT	1.91 ± 3.02	1.44 ± 1.79	0.983
API	51.9 ± 16.4%	52.1 ± 16.7%	0.980
Gingivitis	3 (8.8%)	7 (15.6%)	0.502
Bruxism	19 (55.9%)	25 (55.6%)	1.000
Orthodontic treatment:			
• ongoing	2 (5.9%)	8 (17.8%)	
• needed	4 (11.8%)	2 (4.4%)	0.182
• not needed	28 (82.3%)	35 (77.8%)	
Tooth brushing			
• ≥ 2x/day	30 (88.2%)	40 (88.9%)	
• 1x, only in the morning	1 (2.9%)	3 (6.7%)	0.399
• 1x, only in the evening	3 (8.8%)	1 (2.2%)	
• none	0 (0%)	1 (2.2%)	
Fluoride-containing toothpaste used			
• 500 ppm	1 (2.9%)	1 (2.2%)	
• 1000–1490 ppm	32 (94.2%)	42 (93.3%)	0.971
• no answer	1 (2.9%)	2 (4.4%)	
Knowledge of fluoride gel			
• yes	18 (52.9%)	25 (55.6%)	0.824
• no	16 (47.1%)	20 (44.4%)	
Usage of fluoride gel			
• yes	3 (8.8%)	8 (17.8%)	0.335
• no	31 (91.2%)	37 (82.2%)	

**Fig. 1:**
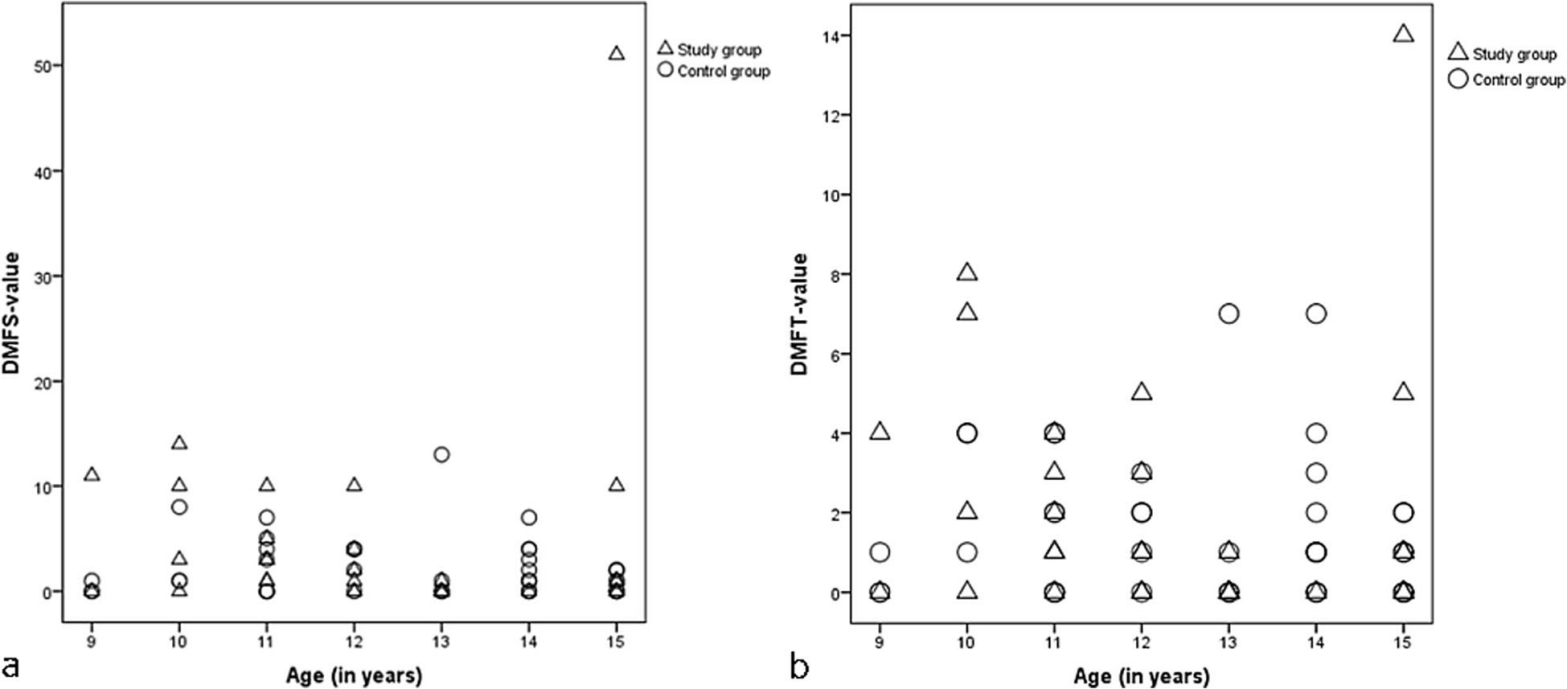
Scatterplots of (**a**) DMFS values (D = decayed, M = missing, F = filled, S = surfaces) and (**b**) of DMFT values (D = decayed, M = missing, F = filled, T = teeth) in relation to age (in years) of children and adolescents with ADHD (Δ; *n* = 34) and of children and adolescents without ADHD (Ο; *n* = 45)

However, differences for DMFS and DMFT values were found when the two groups were analysed separately for the various age groups (9 to 15 years). The scatterplot presented in Fig. 1a shows that participants of the ADHD group tended to have in relation to their age higher DMFS values than those from the control group except for the 13- and 14-year-old children. The scatterplot in Fig. 1b shows that participants with ADHD also tended to have in relation to their age higher DMFT values than those without ADHD except for the 13- and 14-year-old children. When the D and F components were analysed separately it was found that there was no significant difference in mean DT scores between the two groups: DT 0.62 ± 1.18 (ADHD group) versus 0.67 ± 1.21 (control group). In contrast, it was observed that the mean FT scores tended to be higher in the ADHD group (FT 1.06 ± 1.72) than in the control group (FT 0.78 ± 1.19). Therefore, the higher DMFT scores have to be attributed to the higher F component.

The assessment of oral hygiene showed that the ADHD group had a mean API of 51.9 ± 16.4% and the control group had a mean API of 52.1 ± 16.7% (Table 2). Although there was no statistically significant difference between the two groups (*p* = 0.980), the percentages are high in both groups and reflect an oral hygiene which is at best fair and needs to be improved.

Gingivitis was diagnosed only in a small number of participants, in three (8.8%) with ADHD and in seven (15.6%) in the control group (Table 2), whereas the majority had healthy gingival tissues. The difference between the two groups was not statistically significant (*p* = 0.502).

The presence of bruxism was similar in both groups (*p* = 1.000); in the ADHD group, 19 participants (55.9%) had bruxism, and in the control group were 25 participants (55.6%) with bruxism (Table 2). Orthodontic treatment needs differed between participants with ADHD and without ADHD (Table 2; *p* = 0.182). Only two participants in the ADHD group (5.9%) had ongoing orthodontic treatment and four of them (11.8%) needed orthodontic treatment. In contrast, eight participants (17.8%) in the control group received ongoing orthodontic treatment and only two of them (4.4%) needed orthodontic treatment. Both in the ADHD group and in the control group the majority did not need any orthodontic treatment: 28 participants (82.3%) in the ADHD group, and 35 participants (77.8%) in the control group.

### Oral hygiene habits

The results concerning oral hygiene habits are shown in Table 2 and no statistically significant differences were found between the two groups. The majority of the participants in both groups brushed their teeth twice or more often every day (88.2% from the ADHD group and 88.9% from the control group; *p* = 0.399). Tooth brushing only in the morning reported one individual from the ADHD group (2.9%) and three from the control group (6.7%); and tooth brushing only in the evening was practiced by three participants in the ADHD group (8.8%) and one from the control group (2.2%). No tooth brushing was only reported by one person from the control group (2.2%).

Fluoride-containing toothpastes with 1000–1490 ppm fluoride were used by almost all of the participants (94.2% of the ADHD group and 93.3% of the control group; *p* = 0.971). In both groups, only one participant used fluoride-containing toothpaste with 500 ppm (2.9% of the ADHD group and 2.2% of the control group). One participant of the ADHD group (2.9%) and two of the control group (4.4%) gave no answer which toothpaste they used. Fluoride gel was known to 52.9% of the ADHD group and to 55.6% of the control group (*p* = 0.824). However, most of the participants did not use fluoride gel (91.2% of the ADHD group and 82.2% of the control group; *p* = 0.335).

### Dietary habits

Dietary habits in the ADHD group and the control group are summarized in Table 3 and no statistically significant differences were found between the two groups. The majority of the participants drank acidic/sugary beverages (85.3% of the ADHD group and 93.4% of the control group); water alone was consumed only by two participants (5.9%) of the ADHD group and by two (4.4%) of the control group (*p* = 0.390). Frequent intake of acidic/sugary beverages (at least more than 3 times per week to twice or three times daily) was reported more often (41.4%) in the ADHD group versus 33.3% in the control group (*p* = 0.348).

**Table 3:** Dietary habits of the study population

		Children and adolescents with ADHD (study group)	Children and adolescents without ADHD (control group)	*P*-value
Preferred beverage	• water	2 (5.9%)	2 (4.4%)	0.390
	• acidic/sugary beverage	29 (85.3%)	42 (93.4%)	
	• no answer	3 (8.8%)	1 (2.2%)	
Frequency of intake of acidic/sugary beverage	• at least >3 times per week to ≥2x daily	12 (41.4%)	14 (33.3%)	0.348
	• 1x per week	7 (24.1%)	16 (38.1%)	
	• 1x per month	3 (10.3%)	1 (2.4%)	
	• no answer	7 (24.1%)	11 (26.2%)	
Preferred sweets eaten between meals	• chocolate, candy bars, cookies, ice cream	11 (39.3%)	13 (33.3%)	0.378
	• jelly sweets, sour sweets	5 (17.9%)	15 (38.5%)	
	• hard candy, chewing gum	6 (21.4%)	5 (12.8%)	
	• more than one type of sweets	6 (21.4%)	6 (15.4%)	
Frequency of intake of preferred sweets	• daily	11 (40.7%)	10 (25.6%)	0.291
	• 2-3x per week	6 (22.2%)	15 (38.5%)	
	• 1x per week	10 (37.0%)	14 (35.9%)	

Similar numbers of participants from the two groups reported that they consumed sweet snacks: 28 (82.4%) from the ADHD group and 39 (86.7%) from the control group (*p* = 0.378). The preferred sweets eaten between meals and frequency of intake are listed in Table 3. Consumption of chocolate, candy bars, cookies or ice cream was similar in the two groups: 39.3% of the ADHD group and 33.3% of the control group. In contrast, jelly sweets and sour sweets were eaten by fewer participants of the ADHD group (17.9%), whereas in the control group more than twice as many (38.5%) consumed this type of sweets. Hard candy and chewing gums were preferred by 21.4% of the ADHD group, and only by half as many participants (12.8%) of the control group. More than one type of sweets was eaten by 21.4% of the ADHD group and by 15.4% of the control group.

The frequency of intake of preferred sweets differed between the two groups (*p* = 0.291). In particular, daily consumption of sweet snacks was reported more often by participants of the ADHD group (40.7%) and by only 25.6% of the control group. In the ADHD group, 22.2% consumed sweet snacks two or three times weekly, and in the control group 38.5%. Intake of sweet snacks once a week was reported by 37.0% of the ADHD group and by 35.9% of the control group.

### Last dental visit

**Fig. 2:**
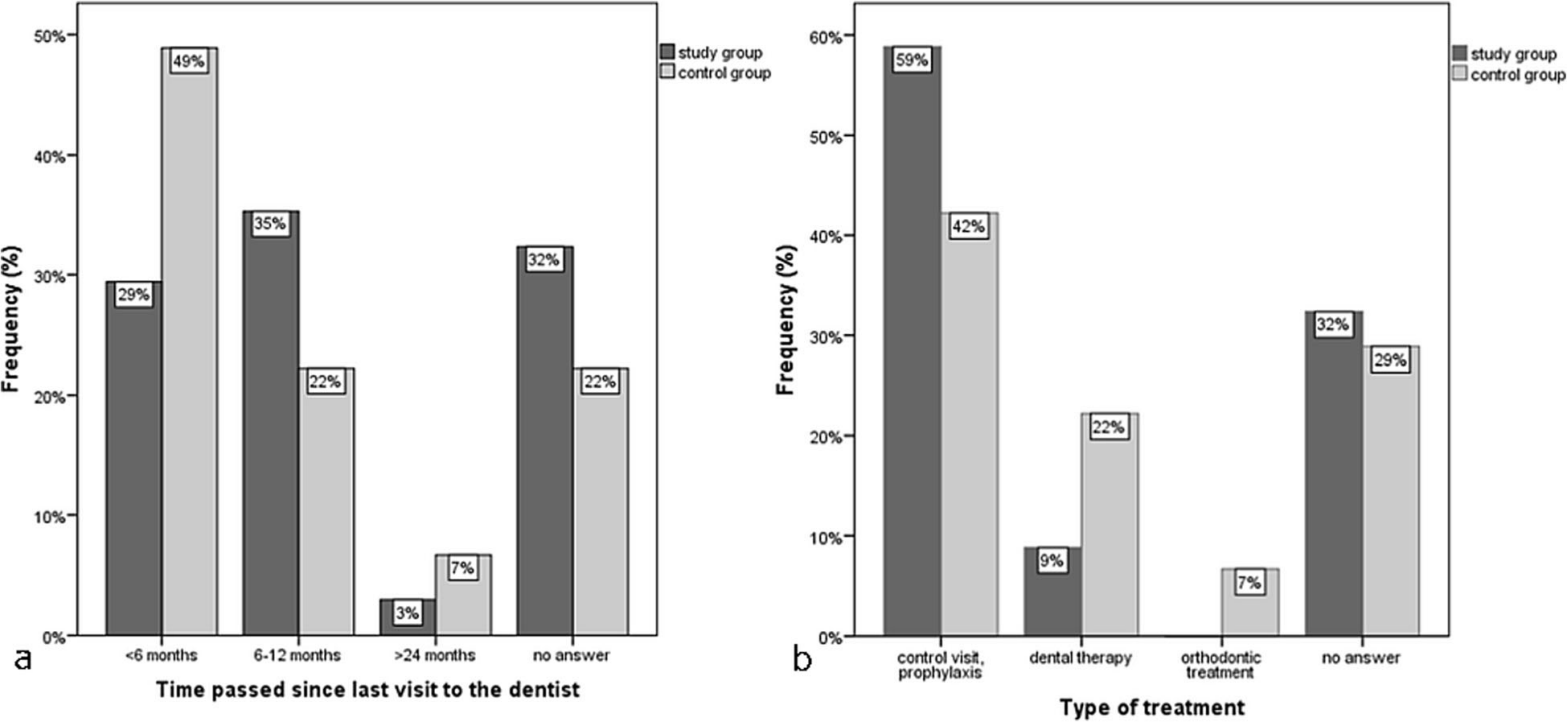
Percentage of children and adolescents with ADHD (study group; *n* = 34) and without ADHD (control group; *n* = 45) (**a**) according to the time passed since their last dental visit and (**b**) according to the type of treatment performed at their last dental visit. Dental therapy consisted of fillings, extractions and endodontic treatments

In the ADHD group, only 29% reported that their last visit to the dentist was less than 6 months ago, whereas in the control group almost half of the participants (49%) had their last visit in this time span (Fig. 2a). Thirty-five percent of the ADHD group and 22% of the control group answered that 6 up to 12 months had passed after their last dental visit. More than a year since their last dental visit had passed for 3% of the ADHD group and 7% of the control group. In the ADHD group 32% gave no answer concerning the time passed since their last visit to the dentist, in the control group it was 22%.

The number/percentage of participants who reported that a treatment was performed during their last dental visit was similar (23/68% in the ADHD group, 29/64% in the control group). However, differences were seen between the two groups concerning the type of treatment (Fig. 2b), but they were not statistically significant (*p* = 0.065). In the ADHD group, the majority of the participants (59%) reported that it was only a control visit or that they received prophylactic measures, whereas dental therapy, which consisted of fillings, extractions and endodontic treatments, was performed on only 9% of the participants. In contrast, only 42% of the control participants had solely gone for a control visit or received prophylactic measures, whereas dental therapy was performed in 22% of the control participants, which was more than twice as many as in the ADHD participants. Orthodontic treatment was only performed on control participants (7%).

## Discussion

This study focussed on the oral health of children and adolescents with or without attention deficit hyperactivity disorder (ADHD) living in residential care. The null hypothesis that oral health parameters of children and adolescents with or without ADHD, living in the same residential care setting under similar conditions, would not be different, was accepted. No statistically significant difference in oral health of children and adolescents with or without ADHD living in residential care was found regarding dmft, DMFT, DMFS, API, bruxism and oral hygiene habits. In spite of these findings, participants with ADHD tended to have higher DMFS/DMFT values than the control group, the ADHD group tended to consume cariogenic drinks and food more often than the controls, and dental and orthodontic treatment was performed less often than in the control group.

More boys than girls seemed to be affected with ADHD with a ratio of 4:1 (27 males, 7 females), which was close to Grooms et al. [17] with 31 males and 7 females, and Chau et al. [7] with 27 males and 4 females in their ADHD groups, and higher than Rowland et al. [22], who found a prevalence ratio of 3:1. A greater proportion of boys having ADHD than girls was observed by another study [23].

There was no statistically significant difference in the dmft/DMFT between the ADHD and non-ADHD groups, which is in agreement with the study by Hidas et al. [8] and Chau et al. [7]. Our participants had a DMFT ≤5, which was, in contrast to the study by Broadbent et al. [23], below their chosen cut-point, where the vast majority of the children with ADHD had a DMFT ≥5. In our study, the DMFS was different between the two groups, but the difference was not significant. This was also found in earlier studies [9, 17]. However, when the DMFS and DMFT scores were analysed separately for the various age groups from 9 to 15 years, the ADHD group tended to have higher values, with the exception of the 13- and 14-year-old children. These results confirm studies by Blomqvist et al. [5, 13, 18]. We found that the 9- to 12-year-old children had a tendency of higher caries experience than those without ADHD. This is in accordance with the study by Blomqvist et al. [5], who also found higher caries experience in 11-year-old children with ADHD than in healthy children; however, the difference was statistically significant. In our study, the 13- to 14-year-old children had in both groups a similar caries experience, which confirms the results of another study by Blomqvist et al. [13]. At the age of 15 the participants with ADHD from our study had again a higher caries experience than the control group. Blomqvist et al. [18] observed in another study with 17-year-old participants again a statistically significant higher caries experience in the ADHD group. According to Maupome [24] at the age of 13 ADHD might be a protective factor against dental caries in spite of bad self-care and dietary patterns. However, at that particular age the recently erupted teeth have not been long enough in situ for caries lesion to develop [13].

The percentages of API were high in both groups, although there was no statistically significant difference between the two groups. This is similar to the findings of Chau et al. [7]. In contrast, earlier studies [8, 9] found statistically significant differences in plaque indices between ADHD and non-ADHD children. In spite of these statistically significant differences in plaque indices, only Chandra et al. [9] observed in addition statistically significantly poorer oral hygiene habits in ADHD children than in the control group, whereas Hidas et al. [8] found no statistically significant differences between ADHD and control group, which is in agreement with our study. Tooth brushing frequencies did not differ between the two groups in the present study, and was similar to other studies [8, 17]. In contrast, it was observed that ADHD children [9] or children who have a risk for conduct/oppositional disorders [14] brushed their teeth statistically significantly less often than children from the control group. The similar tooth brushing habits in our study can be explained by the fact that all children in residential care were supervised by their guardians. Although the majority of the participants reported that they brushed their teeth twice daily or more often, the high values for the API, reflecting only fair oral hygiene, show the need for considerable improvement, including better instructions of guardians and parents.

The percentage of participants consuming acidic/sugary beverages and sweet snacks was similar in the two groups. But regarding frequency of intake of acidic/sugary beverages and sweet snacks, a higher percentage of children from the ADHD group tended to consume these drinks and foods more often than those from the control group. Blomqvist et al. [13] reported also a trend for a higher percentage of children from the ADHD group to frequently eat snacks between meals than in the control group. Chandra et al. [9] found also a higher percentage of children from the ADHD group to frequently eat sweet snacks between meals than in the control group; however, the difference was statistically significant. The study of Dursun et al. [14] confirmed the positive correlation of hyperactivity/inattention scores with an increase in consumption of cariogenic food. The lack of statistical significance in the present study might be explained by the residential care setting, where the children of both groups consume their meals together supervised by guardians.

The presence of bruxism was similar in both groups, which is in agreement with Hidas et al. [8], whereas Chau et al. [7] found a statistically significant higher percentage of bruxism in children with ADHD. There was less ongoing orthodontic treatment and more orthodontic treatment needed in the ADHD group than in the control group. This might be explained by the therapeutic difficulties encountered with children affected by ADHD, as was reported in a study where children with ADHD, due to a short attention span and lack of cooperation, presented more challenges during an orthodontic treatment compared to control participants [25].

A higher percentage of children with ADHD reported only for a dental check-up or received prophylactic measures, whereas actual dental therapy was performed more often in the control participants. Aminabadi et al. [6] observed that children with oppositional defiant disorder (ODD)/ADHD displayed high values of dental anxiety and behaviour-management problems during dental treatment, which could explain the lack of adequate dental therapy in ADHD children.

Due to an unequal sample size the participants with ADHD in the present study were not divided into medicated and non-medicated patients, because the majority (76.5%) was under pharmacotherapy and only eight out of 34 ADHD children received no medication. However, this non-discrimination into medicated and non-medicated participants may have affected the results of the present study. Studies [6, 17, 23] have reported that children with ODD/ADHD had a higher risk of caries than healthy controls, with statistically significantly higher DMFT scores due to their medication [6] or with statistically significantly higher DMFS scores [17]. In children with ODD/ADHD a higher caries risk was found in those under pharmacotherapy compared to those under neuro-feedback therapy with statistically significantly higher DMFT scores [12]. In addition, the plaque index was statistically significantly higher in ODD/ADHD children taking medication than in ODD/ADHD children under neuro-feedback therapy [12]. In the present study, high DMFT scores and high approximal plaque indices were found not only in the participants with ADHD, but also in the controls, all living in the same residential care.

One of the limitations of the present study is the relatively small sample size of 79 participants, but which is similar to the study of Chau et al. [7]. Only simplified examinations were conducted on site in rooms of the residential care setting and no recent dental radiographs were available and no new ones could be taken; thus, it was not possible to reliably identify non-cavitated lesions. Moreover, only absence or presence of gingivitis could be recorded, but clinical examinations such as assessment of gingival bleeding index (GBI) and salivary flow rate could not be conducted due to low compliance and short attention span of the ADHD participants. Earlier studies [5, 6, 13] have shown no statistically significant differences in GBI [6, 13] and in saliva production [17] between ADHD and non-ADHD participants, whereas in other studies statistically significant higher gingival bleeding [7, 18] and lower unstimulated salivary flow rates in children using methylphenidate [12] were found in children with ADHD.

The findings of the present study show the need for considerable improvement of oral hygiene and dietary habits in children and adolescents, who live in residential care. In children with ADHD, the most effective method of reducing dental caries seems to be more frequent dental visits with a) instructions for better oral hygiene at home and b) dietary counselling to reduce the consumption of sweet snacks and drinks, including parents as well as guardians [26]. In particular, the guardians in this residential care center, who were specially trained to care for children and adolescents with ADHD, need to be further instructed in better supervision of oral hygiene practices. They also need to be alerted to paying more attention to healthier eating and drinking habits, possibly with the assistance of a dietician, from which in consequence all children would benefit. In addition, dental and orthodontic therapy was performed less often in the children and adolescents with ADHD. Referrals to specially trained dentists and orthodontists might help children with ADHD to get the appropriate dental treatment they actually need. More awareness among clinicians to facilitate better caries- and trauma-preventive management is also important [27].

## Conclusions

The findings of the present study show that there was no statistically significant difference in oral health of children and adolescents with or without ADHD living in residential care. However, ADHD participants had higher DMFT/DMFS values and consumed more cariogenic drinks and food. In addition, ongoing orthodontic treatment was performed less often in these participants. Parents and guardians need better instructions for a more sufficient supervision of oral hygiene and dietary habits in children and adolescents with or without ADHD.

## Supplementary information

**Additional file 1.** Questions concerning oral hygiene and dietary habits.

## Data Availability

The datasets generated and/or analysed during the current study are not publicly available due to guarantee anonymity of the participants, but are available from the corresponding author on reasonable request.

## Abbreviations

ADHD: Attention deficit hyperactivity disorder
API: Approximal plaque index
DMFS/DMFT: Decayed, missing, filled surfaces/teeth in the secondary dentition
dmft: Decayed, missing, filled teeth in the primary dentition
GBI: Gingival bleeding index
ICD-10: International Statistical Classification of Diseases and Related Health Problems 10th Revision
IQ: Intelligence quotient
max: Maximum
min: Minimum
ODD: Oppositional defiant disorder
OH: Oral health score
SD: Standard deviation
