# Supplementary material for: Oral health of children and adolescents with or without attention deficit hyperactivity disorder (ADHD) living in residential care in rural Rhineland-Palatinate, Germany
Source: BMC Oral Health. 2019 Nov 25;19:258. doi: 10.1186/s12903-019-0948-5 (PMC6878625; doi:10.1186/s12903-019-0948-5)
Supplement: Supplementary file 1 — Additional file 1. Questions concerning oral hygiene and dietary habits. [file 12903_2019_948_MOESM1_ESM.doc]

**Questionnaire**

Oral hygiene habits

| 1. When do you brush your teeth? | 🗌  in the morning | 🗌  at noontime | | 🗌  in the evening | | 🗌 |
| --- | --- | --- | --- | --- | --- | --- |
| 1. When do you forget to do it? | 🗌 in the morning | | 🗌 at noontime | | 🗌 in the evening | |
| 1. How often? |  | | | | | |
| 1. Which toothpaste do you use? |  | | | | | |
| 1. Do you know fluoride gel? | 🗌 yes | | | 🗌 no | | |
| 1. Do you use it? | 🗌 yes | | | 🗌 no | | |
| 1. When did you last visit a dentist? |  | | | | | |
| 1. What did he do? |  | | | | | |
| 1. Have you ever had a toothache? |  | | | | | |
| 1. What did you do then? |  | | | | | |

Dietary habits

| 1. What beverage do you like to drink? |  |
| --- | --- |
| 1. When do you drink it?   How often per day?  How often per week? |  |
|  |
|  |
| 1. When do you drink water? |  |
| 1. How many glasses? |  |
| 1. Which sweets do you like? |  |
| 1. When do you eat them? |  |
| 1. How often do you eat sweets? |  |
| 1. Which snack do you eat in the   morning? |  |
| 1. Which snack do you eat in the   afternoon? |  |
